# Calculated Drug Concentrations in Currently Available Intravitreal Therapies: Determination of Dilution Factor and Deviation From Recommended Doses

**DOI:** 10.7759/cureus.65888

**Published:** 2024-07-31

**Authors:** Andreas F Borkenstein, Eva-Maria Borkenstein, Armin Presser

**Affiliations:** 1 Ophthalmology, Borkenstein and Borkenstein, Private Practice at Privatklinik Kreuzschwestern Graz, Graz, AUT; 2 Pharmaceutical Chemistry, Institute of Pharmaceutical Sciences, University of Graz, Graz, AUT

**Keywords:** recommended dose, dilution factor, drug concentration, vitreous volume, diabetic macular edema, age-related macular degeneration, intravitreal injection, intravitreal therapy

## Abstract

In ophthalmology, intravitreal therapies are currently not personalized/customized and are not adjusted to the individual vitreous volume. With reference to the recently published calculation formula for a more accurate estimation of the vitreous body, we determined the dose of intravitreal medication for different vitreous volumes and compared them with the average volume. Using the axial length of the eye, the formula for the vitreous volume exact (VIVEX) can provide a more accurate indication of the vitreous volume in individual cases than an assumed standard volume of 4 mL. The concentration of active substances in small eyes may be twice as high as that in normal-sized emmetropic eyes. In contrast, large eyes may show less than half of the recommended drug concentration. The calculated concentrations of the investigated intravitreal drugs in small and large eyeballs showed impressive differences with large deviations from the recommended doses. Further systematic studies should follow to find out whether this has any impact on the effectiveness or side effects of the injected drugs.

## Introduction

Personalized medicine (PM) is based on the understanding of individual differences between patients and represents a renunciation of the concept of "one size fits all" [1,2]. According to this concept, the most appropriate therapy should be selected for a specific individual to optimize effects and minimize side effects. In pharmacology and medicine, quantities and dosages are adjusted individually. Many drugs are dosed according to body weight (mg/kg) or body surface area (mg/m²). The principle "as much as necessary, as little as possible" applies in various areas of medicine. Moreover, the term PM is also used for treatments that use a person's individual genetic profile as a decision-making tool for the prevention and treatment of diseases. There are also approaches to practice a personalized vaccinology [3].

In ophthalmology, intravitreal therapies and intravitreal injections (so-called IVIs) are currently not personalized/customized and are not adjusted to the individual vitreous volume. This also means that all patients with differently-sized eyes currently receive exactly the same intravitreal dosage of medication. Therefore, the main question is “Can we also apply the concept of PM to intravitreal therapies in ophthalmology?”

Intravitreal drug deliveries have revolutionized the treatment of many retinal diseases, including neovascular age-related macular degeneration (AMD), diabetic retinopathy (DR), and retinal vein occlusion [4,5]. Moreover, diseases like uveitis, endophthalmitis, and cystoid macular edema are common diseases that are treated by intravitreal injections [6]. AMD is a leading cause of vision loss and blindness worldwide. The worldwide prevalence of AMD is reported to be 8.7% among people aged between 45 and 85 years. Forecasts are that 288 million people worldwide will be affected by AMD in 2040 [7-9]. DR is a common microvascular complication of diabetes and the leading cause of blindness among working-age adults in the United States [10,11].

There are guidelines for the selection of intravitreal agents and treatment recommendations from experts for an optimal approach [4]. Ganciclovir was the first widely available, relatively inexpensive, compounded drug used for repeated intravitreal injections to treat chronic retinal disease, followed by triamcinolone for diabetic macular edema and bevacizumab for neovascular AMD. A large number of drugs are now administered by intravitreal injection; some are not yet authorized but are used off-label.

According to market research data and the American Academy of Ophthalmology (AAO) News/Health Statistics, it is estimated that nearly seven million intravitreal injections are performed in the United States annually, making it one of the most commonly performed procedures in all of medicine [4,5]. The global intravitreal injectable (IVI) market valued at USD 17,559.91 million in 2023 is projected to reach USD 26,520.45 million by 2032, reflecting a compound annual growth rate (CAGR) of 4.16% [12].

It should be noted at this point that there have been dosage recommendations for these drugs since the approval studies. However, these are currently not adjusted to the actual vitreous body volume. Each eye receives the same dose, regardless of the size of the vitreous body volume. Pivotal studies mostly used consecutive cases with the corresponding diseases based on inclusion or exclusion criteria, but no systematic studies and special consideration were given to anatomical differences, such as very small eyeballs versus very large eyeballs.

Recently, studies using magnetic resonance imaging (MRI) and biometric measurements of the anatomy of the eyes, including anterior chamber depth, lens thickness, and axial length of the eyeball, have shown that there are impressive differences in the size ratios of the vitreous body volume [13]. The specification of a “standard volume” of 4-5 mL in literature and many books on ophthalmology appears to be inaccurate and outdated. It has been shown that hyperopic, small eyes have vitreous volumes of 2-3 mL, and highly myopic, long eyes have large vitreous volumes of >9-10 mL, which is four times the volume of a hyperopic eye. The authors also introduced a novel calculation formula (vitreous volume exact (VIVEX)) to determine the vitreous body volume more accurately based on the axial length of the eye [13]. This can be done quickly and easily by measuring the axial length of the eye using ultrasound or modern biometric devices during a routine ophthalmological examination. The value of the axial length is then used as a parameter in the calculation formula to obtain a more accurate vitreous volume.

## Technical report

With reference to the anatomical spherical shape with the diameter of the axial length (AL), which has a volume of AL^3^·π/6, a correction element of 0.76 + 0.012·(AL-24) was derived (VIVEX formula) [13].

$V=\frac{{\rm AL}^3\bullet\pi}{6}\bullet(0.76+0.012\bullet(AL-24))$

This is necessary to take into account the proportion of the vitreous body and the proportional enlargement of the vitreous body in long (myopic) eyes.

**Figure 1:**
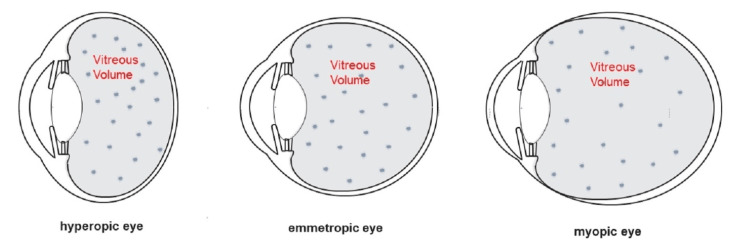
Schematic representation of different drug concentrations according to different vitreous volumes in small, hyperopic eyes, emmetropic eyes, and large, myopic eyes Image credits: Andreas F. Borkenstein and Armin Presser.

Using this correction formula, typical vitreous volumes were calculated in currently available intravitreal therapies for small, hyperopic eyes (2-3 mL), emmetropic eyes (4-5 mL), and large, myopic eyes (9-10 mL). Referring to the currently recommended doses of intravitreal drugs at the standard volume, the concentrations for different vitreous body volumes were calculated. We included anti-VEGFs, complement inhibitors, recombinant proteases, anti-infective drugs, and corticosteroids. The dilution effect and the deviation from the recommended dose in a specific vitreous volume immediately after application were analyzed. Schematic eye models were used to visualize the anatomical differences (Figure 1).

In Tables 1, 2, the dilution factor and the deviation from the recommended default dosage were calculated by referring to common ophthalmic drugs. It was found that the concentration of active substances in rather small, hyperopic eyes is twice as high as in normal-sized emmetropic eyes. In contrast, for large, myopic eyes, less than half of the recommended drug concentration is achieved. Since the drug dilution factor (DDF) and the deviation from the recommended dose (DRD) are unrelated to the specific drug and depend only on the individual vitreous volume, the corresponding values are identical.

**Table 1:** Calculated drug concentration of common anti-VEGF therapeutics in the vitreous immediately after application of the recommended dose as a function of the vitreous volume. The values for average-sized eyes are indicated in bold. ^a^ Drug dilution factor (DDF) in relation to the volume of an “average,” emmetropic eye with 4 mL. ^b^ Deviation from the recommended dose (DRD). VEGF: Vascular endothelial growth factor.

Vitreous volume	Aflibercept	Bevacizumab	Brolucizumab	Faricimab	Ranibizumab	DDF^a^	DRD^b^
2 mL	1.000 mg/mL	0.625 mg/mL	3.000 mg/mL	3.000 mg/mL	0.250 mg/mL	2.00	200%
3 mL	0.667 mg/mL	0.417 mg/mL	2.000 mg/mL	2.000 mg/mL	0.167 mg/mL	1.33	133%
4 mL	0.500 mg/mL	0.313 mg/mL	1.500 mg/mL	1.500 mg/mL	0.125 mg/mL	1.00	100%
5 mL	0.400 mg/mL	0.250 mg/mL	1.200 mg/mL	1.200 mg/mL	0.100 mg/mL	0.80	80%
6 mL	0.333 mg/mL	0.208 mg/mL	1.000 mg/mL	1.000 mg/mL	0.083 mg/mL	0.67	67%
7 mL	0.286 mg/mL	0.179 mg/mL	0.857 mg/mL	0.857 mg/mL	0.071 mg/mL	0.57	57%
8 mL	0.250 mg/mL	0.156 mg/mL	0.750 mg/mL	0.750 mg/mL	0.063 mg/mL	0.50	50%
9 mL	0.222 mg/mL	0.139 mg/mL	0.667 mg/mL	0.667 mg/mL	0.056 mg/mL	0.44	44%
10 mL	0.200 mg/mL	0.125 mg/mL	0.600 mg/mL	0.600 mg/mL	0.050 mg/mL	0.40	40%

**Table 2:** Calculated drug concentration of other ophthalmic therapeutics in the vitreous immediately after application of the recommended dose as a function of the vitreous volume. The values for average-sized eyes are indicated in bold. ^a ^Drug dilution factor (DDF) in relation to the volume of an “average,” emmetropic eye with 4 mL. ^b ^Deviation from the recommended dose (DRD).

Vitreous volume	Ceftazidime	Methotrexate	Pegcetacoplan	Triamcinolone acetonide	Vancomycin	DDF^a^	DRD^b^
2 mL	1.125 mg/mL	0.200 mg/mL	7.500 mg/mL	2.000 mg/mL	0.500 mg/mL	2.00	200%
3 mL	0.750 mg/mL	0.133 mg/mL	5.000 mg/mL	1.333 mg/mL	0.333 mg/mL	1.33	133%
4 mL	0.563 mg/mL	0.100 mg/mL	3.750 mg/mL	1.000 mg/mL	0.250 mg/mL	1.00	100%
5 mL	0.450 mg/mL	0.080 mg/mL	3.000 mg/mL	0.800 mg/mL	0.200 mg/mL	0.80	80%
6 mL	0.375 mg/mL	0.067 mg/mL	2.500 mg/mL	0.667 mg/mL	0.167 mg/mL	0.67	67%
7 mL	0.321 mg/mL	0.057 mg/mL	2.143 mg/mL	0.571 mg/mL	0.143 mg/mL	0.57	57%
8 mL	0.281 mg/mL	0.050 mg/mL	1.875 mg/mL	0.500 mg/mL	0.125 mg/mL	0.50	50%
9 mL	0.250 mg/mL	0.044 mg/mL	1.667 mg/mL	0.444 mg/mL	0.111 mg/mL	0.44	44%
10 mL	0.225 mg/mL	0.040 mg/mL	1.500 mg/mL	0.400 mg/mL	0.100 mg/mL	0.40	40%

**Figure 2:**
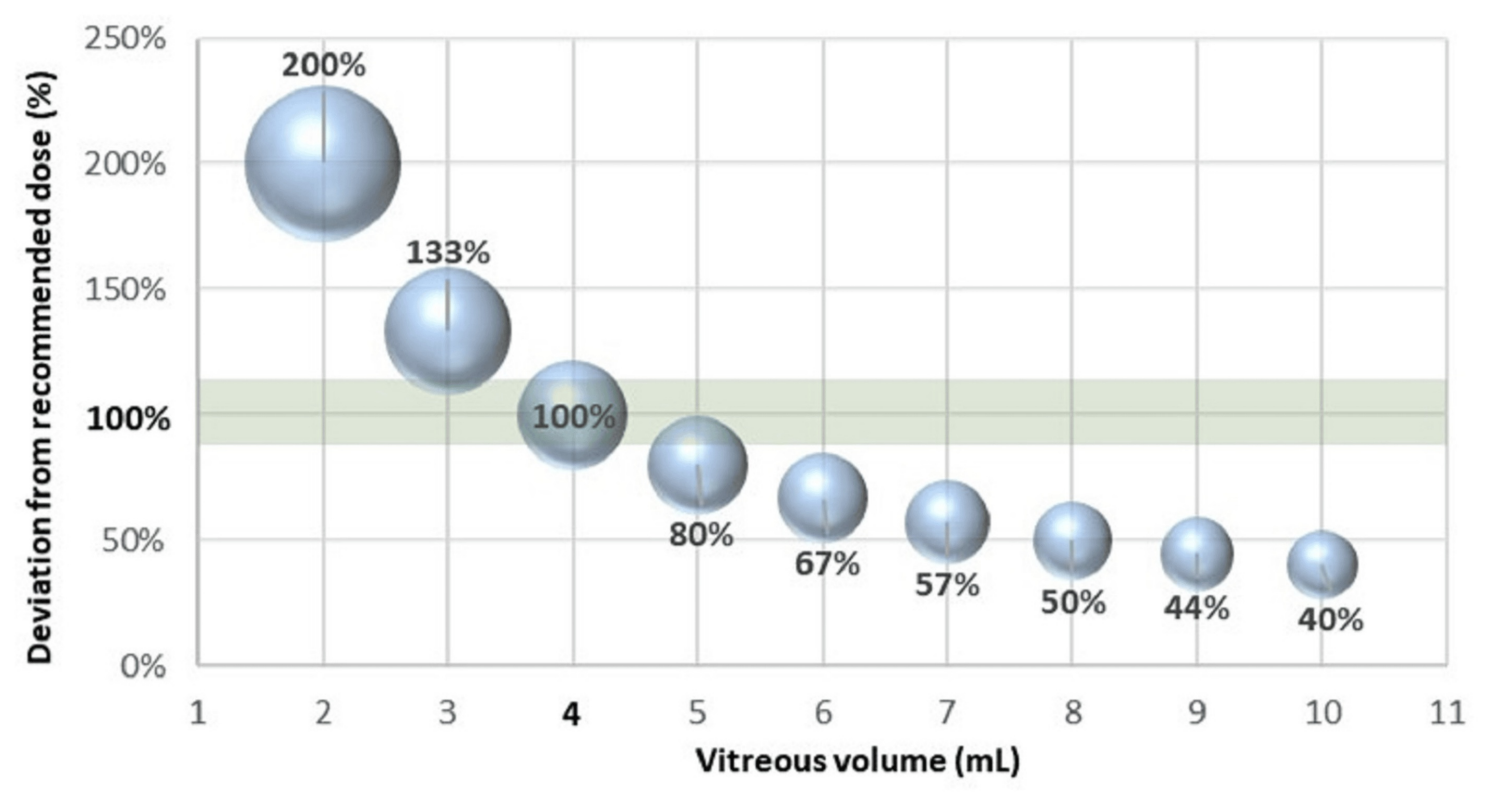
Schematic visualization of the large, calculated concentration differences after injection of the recommended dose into the vitreous body of different sizes (the target area is shaded). Image credits: Andreas F. Borkenstein and Armin Presser.

Figure 2 visualizes the large differences in drug concentration for different-sized vitreous bodies. It is known that the appropriate dosage and concentration of the active agents also play a crucial role in intraocular injections [14,15]. However, to our knowledge, there are currently no available data on whether and how such deviations from the recommended drug concentration actually affect medical treatment, nor are there any recommendations to adjust the dose to the vitreous body volume.

## Discussion

The huge increase in intravitreal injections administered worldwide is largely driven by the introduction of new drug therapies and the expansion of their indications. A variety of drugs, including anti-VEGF agents, corticosteroids, and neuroprotective drugs, have shown promising results in clinical trials [16-18]. However, the actual concentration of active ingredients in the vitreous should not be underestimated at all. Frequently used agents in intravitreal therapies are shown in Table 3.

**Table 3:** List of frequently used ophthalmological therapeutics and their recommended dosage

Drug	Common dose
*Anti-VEGF drugs, complement inhibitors, and recombinant proteases*	
Aflibercept (Eylea^®^)	2.0 mg/0.05 mL [19]
Bevacizumab (Avastin^®^)	1.25 mg/0.05 mL (off-label) [20]
*Bevacizumab *gamma (Lytenava^®^)	1.25 mg/0.05 mL [21]
Brolucizumab (Beovu^®^)	6.0 mg/0.05 mL [22]
Faricimab (Vabysmo^®^)	6.0 mg/0.05 mL [23]
Ocriplasmin (Jetrea^®^)	0.125 mg/0.1 mL [24]
Pegcetacoplan (Syfovre^®^)	15 mg/0.1 mL [25]
Ranibizumab (Lucentis^®^)	0.5 mg/0.05 mL [26]
*Anti-infective drugs*	
Amphotericin B	0.005 mg/0.1mL [27]
Ceftazidime	2.25 mg/0.1 mL [20]
Clindamycin	1.0 mg/0.1 mL [28]
Foscarnet	2.4 mg/0.1 mL [20]
Ganciclovir	6 mg/0.1 mL [29]
Vancomycin	1.0 mg/0.1 mL [20]
*Corticosteroids*	
Dexamethasone	0.4 mg/0.1 mL [30]
Triamcinolone acetonide (Triesence^®^)	4 mg/0.1mL [31]
*Drugs for vitreoretinal lymphoma*	
Methotrexate	400 μg/0.1 mL [20]

We emphasize that currently available intravitreal therapies are safe and effective and have led to good clinical results in recent years, as demonstrated by countless clinical studies worldwide.

Our considerations and theoretical experiments should therefore not call this into question. Our goal is to highlight the fact that eyes have large differences in anatomical dimensions. The option to use a calculation formula to get a more accurate dose according to the individual vitreous volumes is easy and fast. Now, it could be possible to carry out systematic investigations depending on the vitreous body volume.

Systematic studies should compare the effect and side effect profiles of the drugs with different concentrations in the vitreous cavity. It seems surprising that if the anatomical differences are so great, the same dose (“one size fits all concept”) is the best approach. Rather, it should not be surprising if adapted and individualized approaches could bring astonishing benefits in the future. In the literature, there are various case reports of retinal intoxications following high-dose intravitreal amphotericin B injections [32,33]. Furthermore, other studies showed that higher doses of intravitreal triamcinolone acetonide (IVTA) for diabetic macular edema achieved greater improvements in visual acuity and reductions in retinal thickness than lower doses [34].

Multidisciplinary medicinal and pharmaceutical studies should therefore be carried out to systematically investigate the following questions:

1. Does the addition of a certain drug/solution volume in small, hyperopic eyes (low vitreous volume) lead more frequently to a postoperative increase in intraocular pressure than in large, myopic eyes with a larger vitreous volume?

2. Do myopic eyes with larger vitreous volumes need more frequent re-treatments and does the dilution effect have any impact on the effect or duration of the effect of intravitreal medications?

3. Does the addition of a relatively large volume into a smaller eye lead to a higher risk of any anatomical displacement of the iris-lens diaphragm or more frequent local or systemic side effects?

4. With the knowledge of the high frequency of these intravitreal injections worldwide, could a customized, individualized approach achieve any economic benefits (shorter waiting times and faster treatments) or offer financial advantages?

It is evident that this is far more complex: In addition to the anatomical differences and individual vitreous volumes, many other factors are decisive. Drugs and their mechanisms of action, receptor binding, and responders/non-responders must be differentiated. In any case, it seems logical that personalized dosing of IVIs with adjustment to the anatomical differences and volumes of the vitreous could make it possible to treat patients more effectively in the future.

Limitations

However, there are a few limitations. Personalized and adapted intravitreal therapy would entail considerable additional efforts, including preoperative measurement and determination of the vitreous volume as well as tailored administration of the respective medication. This would considerably increase the time and cost involved. Therefore, systematic, multi-center studies need to be performed in advance to prove whether an individualized approach can actually offer advantages in the form of better efficacy or fewer side effects.

## Conclusions

It was shown that the recently published correction formula (VIVEX) can be used for calculating the individual vitreous body volume more precisely. This means that it will be easier to carry out various systematic studies to better assess the effects and side effects of intravitreal drug applications, taking the individual vitreous body volume into account. After conducting these studies, it will be possible to determine whether a dosage regimen tailored to the individual patient would in fact be beneficial. In any case, this point should be clarified in the context of extensive scientific studies, which is now easier to achieve using the new calculation formula.

We have demonstrated that the effective concentration of a drug in the vitreous can vary significantly according to volume. The calculated concentrations of frequently used drugs in small and large eyeballs showed impressive differences with large deviations from recommended doses.
